# Influence of mTOR-inhibitors and mycophenolic acid on human cholangiocellular carcinoma and cancer associated fibroblasts

**DOI:** 10.1186/s12885-016-2360-8

**Published:** 2016-05-20

**Authors:** Nils Heits, Tillmann Heinze, Alexander Bernsmeier, Jannik Kerber, Charlotte Hauser, Thomas Becker, Holger Kalthoff, Jan-Hendrik Egberts, Felix Braun

**Affiliations:** Department of General, Visceral-, Thoracic-, Transplantation- and Pediatric Surgery, University Medical Center Schleswig-Holstein (UKSH), Campus Kiel, Arnold-Heller-Strasse 3 (Haus 18), 24105 Kiel, Germany; Division of Molecular Oncology, Institute for Experimental Cancer Research, University Medical Center Schleswig-Holstein (UKSH), Campus Kiel, Arnold-Heller Str. 3, 24105 Kiel, Germany

**Keywords:** Cholangiocarcinoma, Cancer associated fibroblast, mTOR-inhibitor, Mycophenolic acid, Tumour growth, Liver transplantation, Tumour migration, Tumour proliferation, Cytokine expression, JAK/STAT-pathway, ERK-pathway, AKT-pathway

## Abstract

**Background:**

The incidence of Cholangiocellular Carcinoma (CCA) is increasing in the western world. The tumour has a high proportion of desmoplastic stroma and is correlated with a worse prognosis when cancer associated myofibroblasts (CAFs) are present. Recent studies showed promising results after liver transplantation (LTx) in non-resectable early stage CCA. Mycophenolic acid (MPA) and the mTor inhibitor Everolimus are used to prevent organ rejection but recently were shown to exhibit an antiproliferative effect on CCA-cells. Little is known about the influence of immunosuppressive drugs on tumour cell proliferation and migration after paracrine stimulation by CAFs. Moreover, it is still unknown, which signaling pathways are activated following these specific cell-cell interactions.

**Methods:**

CCA cell lines HuCCT1 and TFK1 were utilized for the study. CAFs were derived from resected CCA cancer tissue. Cell viability was measured by the crystal violet assay and tumour cell invasion was quantified using a modified co-culture transmigration assay. Semiquantitative cytokine-expression was measured using a cytokine-array. Protein expression and phosphorylation of ERK, STAT3 and AKT was determined by Western-blot analysis.

**Results:**

CCA cells treated with MPA exhibited a dose related decrease in cell viability in contrast to Cyclosporine A (CSA) treatment which had no effect on cell viability. Everolimus significantly inhibited proliferation at very low concentrations. The pro-invasive effect of CAFs in co-culture transmigration assay was significantly reduced by Everolimus at a concentration of 1nM (*p* = 0.047). In contrast, MPA and CSA showed no effect on tumour cell invasion. Treatment of CAFs with 1nM Everolimus showed a significant reduction in the expression of IL 8, IL 13, MCP1, MIF and Serpin E1. CCA-cells showed significant increases in phosphorylation of ERK, STAT3 and AKT under the influence of conditioned CAF-media. This effect was suppressed by Everolimus.

**Conclusions:**

The secretion of proinflammatory cytokines by CAFs may lead to increased activation of JAK/STAT3-, ERK- and AKT-signaling and increased migration of CCA-cells. Everolimus abrogates this effect and inhibits proliferation of CCA-cells even at low concentrations.

LTx for non-resectable early stage CCA is currently performed in several clinical studies. Consistent with a role for common immunosuppressants in inhibiting tumour cell-proliferation and -invasion, our study indicates that a combination of standard therapies with Everolimus and MPA is a promising therapy option to treat CCA following LTx.

## Background

The incidence of cholangiocellular carcinoma (CCA) has been increasing over the past decades [1]. Currently surgical resection is the only curative treatment option. However, in most cases the tumour is non resectable at the time of diagnosis leaving only palliative treatment options which have low survival rates [2–5]. Recently, there has been a renewed interest in performing orthotopic liver transplantation (OLTx) as an alternative approach to treat CCA. Published results from the latest clinical studies have indicated 5-year survival rates between 71 and 82 % for non-resectable early stage CCA [6]. Therefore OLTx has become a feasible treatment option and could offer better survival rates than palliative therapy [7]. In the above mentioned studies the recipients were treated with neo-adjuvant therapy based on the Mayo protocol [8, 9]. In this protocol only patients with locally non-resectable early stage CCA or arising CCA in the setting of underlying primary sclerosing cholangitis (PSC) were included.

The administration of immunosuppressive drugs in cancer patients has generally been avoided due to the suspected risk of tumour progression when supressing the human immune system. However, over the last decade several substances which were classically used as immunosuppressive drugs have elicited beneficial anti-cancer effects. One of the promising agents for mediating immunosuppression and anti-cancer effects following OLTx is rapamycin, which inhibits mTOR protein kinase activity. Activation of mTOR leads to increased tumour progression [10] and expression of pro-angiogenic growth factors [11] by two distinct complexes: mTOR complex 1 (mTORC1) and mTOR complex 2 (mTORC2). Functionally mTORC1 affects cell growth by regulating mRNA translation and ribosome biogenesis and negatively regulates AKT activation. mTORC2 activates AKT and phosphorylation of downstream effectors promotes cell survival, proliferation and metabolism. It has previously been observed that mTOR inhibitors like Rapamycin reduce CCA progression and enhance long-term survival in patients with inoperable CCA [12–15]. A second recently developed mTOR inhibitor, Everolimus, is endowed with a more favourable pharmacokinetic profile [16, 17] and targets primarily mTORC1 inhibiting cell cycle progression, survival, and angiogenesis [18].

The immunosuppressive agent Mycophenolic acid (MPA) is used to prevent acute graft rejection after transplantation. MPA inhibits inosine monophosphate dehydrogenase (IMPDH), which leads to inhibition of de novo synthesis of guanosine nucleotides [19–22]. This is the principle mechanism by which the prodrug of MPA, mycophenolate mofetil (MMF) blocks T and B lymphocyte proliferation and clonal expansion, and prevents the generation of cytotoxic T cells and other effector T cells. Furthermore, several studies showed that IMPDH can function as a sequence-specific DNA-binding transcription factor [23] by binding and repressing histone genes and E2F, the master driver of the G1/S transition of the cell cycle. Since IMPDH and particularly IMPDH2 are significantly up-regulated in many tumour cells, [24, 25] they are potential targets for anti-cancer strategies. Several studies have shown MMF to inhibit cancer cell proliferation and induce apoptosis in vitro and in vivo [26–31]. Mechanisms for this anticancer effect are postulated to be mediated through activation of the key tumour suppressor molecule p53 [32] by IMPDH and its ability to inhibit the surface expression of some integrins [33].

Several studies have reported a strong impact of tumour-stroma interaction and extracellular matrix proteins in the development of CCA. Cancer associated fibroblasts (CAFs) have been shown to be a key player in creating an inflammatory microenvironment which stimulates invasion of tumour cells [34]. Increased immunohistochemical staining of α-smooth muscle actin (α-SMA) in CAFs has been shown to correlate with shorter survival times as well as a larger tumour size in surgically resected intrahepatic CCA [34–36]. Therefore, agents that inhibit or reduce paracrine interactions between CCA tumour cells and CAFs leading to an inhibition of tumour invasion and proliferation can potentially have therapeutic application in anticancer treatment of CCA.

In this study we have examined in vitro, the anticancer properties of the two immunosuppressive agents, Everolimus and MPA. With a view to a possible application of these drugs following OLTx, the effect on CCA tumour cell-proliferation and invasion was compared with the well established immunosuppressive drug Cyclosporine A (CSA). Special focus was given to possible interactions between CAFs and CCA-tumour cells in stimulating tumour cell-proliferation, invasion and a possible effect of the drugs in the inhibition of paracrine interactions.

## Methods

### Cells

The CCA cell-lines HuCCT-1 (intrahepatic/distal tumour) and TFK-1 (extrahepatic/hilar tumour) were used. Cells were obtained from Cell Bank RIKEN Bio Resource Centre in Japan.

CAFs were obtained from tumour resections following patient’s informed consent and the use of patient’s tumour tissue was approved by the local ethics committee of the “Medizinische Fakultät der Christian-Albrechts-Universität zu Kiel” (AZ 110/99). Directly after resection, liver tissue was cut into small pieces and cultured in Dulbecco’s Modified Eagle Media (DMEM). Adherent cells were collected and characterized by immunocytochemical staining for α-SMA, Vimentin and pan-cytokeratin marker. Negative control stained cells were counterstained with hemalaun/eosin.

### Cell growth/viability assay

Cells were seeded into 96-well plates (tumour cells 1×10^4^ cells/well; CAF’s 2,5×10^3^ cells/well) in DMEM supplemented with 10 % FCS. One day later the media was replaced by fresh DMEM plus 10 % FCS containing different concentrations of CSA, Everolimus and MPA. Cell viability was measured after 24, 48 and 96 h using Crystal violet assay and compared to the viability of the non treated tumour cells and CAFs. The calcineurin inhibitor CSA, which is used as a common immunosuppressive drug following OLTx, was used as a reference. The drug was selected as a control, because no inhibitory effect on tumour proliferation and migration was expected.

### Migration assay

Migration of untreated tumour cells, tumour cells co-cultured with CAFs and with MPA, Everolimus and CSA treatment were analysed. The analysis was performed using a modified Boyden chamber assay, using cell culture inserts for 24-well plates containing membranes with 8 μm pore size. In the CAF/tumour cell co-culture, CAF’s were seeded in a density of 3×10^4^cells/well in DMEM in the lower compartment. After overnight attachment, media was replaced and matrigel-coated inserts were added. Afterwards 5×10^4^ tumour cells were seeded in the upper chamber and Everolimus, CSA or MPA were added directly into the medium. An incubation time of 30 h was used to minimize the bias of proliferation in this assay. The examination area for the cell count was 0,35 mm^2^. For further analysis, the cell count of migrated cells without CAFs in a co-culture was set as 1. For comparison of treated and non-treated co-culture groups an index was calculated.

### Western blot analysis

A fluorescent read-out was used to detect drug target proteins for mTOR and calcineurin in both tested cell lines TFK-1 and HUCCT-1 after 24, 48 and 96 h. These time points corresponded to the cell viability measurement after treatment with the tested drugs. To study the effects of CAFs on the JAK/STAT, AKT- or ERK-pathway, conditioned CAF-media in which fibroblasts had grown for 48 h, was added to DMEM-media and compared to the activation of the specific pathways under DMEM-media without CAF-media. The influence of Everolimus on the activity of the JAK/STAT-, AKT- and ERK-pathway was investigated by measuring STAT/pSTAT, AKT/pAKT as well as ERK/pERK for tumour cells that were treated with Everolimus. Specifically, cells were seeded into 6-well plates and incubated for 24 h at a temperature of 37 °C in DMEM supplemented with 10 % FCS or 0 % FCS. One day later the media was replaced by fresh DMEM with or without conditioned CAF-media. To investigate the effect of Everolimus on tumour cells, the drug was incubated in the presence of tumour cells for 24 h. To investigate the paracrine effect of Everolimus, CAFs were treated with 1 μM Everolimus for 24 h prior to stimulation of tumour cells with the Everolimus-treated CAF-media. As a reference, cells were stimulated with 100 ng/ml hIL-6. Cells in 6-well plates were lysed by RIPA-lysis-buffer followed by protein extraction using ultrasound sonication. The protein assay was done by DC-protein assay (Bio-Rad Laboratories©, Munich, Germany). Protein concentrations were adjusted and diluted by RIPA-lysis-buffer. Samples were then loaded in duplicate and separated by SDS-PAGE and transferred to FL-membranes (Novex, Life Technologies, Carlsbad, CA). The membranes were blocked in 5 % BSA in TBS, then incubated with primary antibodies for pSTAT3/STAT3, pERK/ERK, pAKT/AKT, ß-Actin and drug-target protein specific antibodies for mTOR and calcineurin (IRDye® 800 CW Goat anti-Rabbit IgG, IRDye® 680 RD Goat anti-Mouse IgG). The membranes were washed three times for 10 min in TBST and then probed with goat anti-mouse/rabbit IR-Dye 670 or 800cw labelled secondary antisera (LI-COR, Bad Homburg, Germany) for 1 h at room temperature. Membranes were imaged using a LiCOR Odyssey scanner. Regions of interest were manually placed around each band, which returned near-infrared fluorescent values of raw intensity. Intra-lane background values were subtracted using Odyssey 3.0 analytical software (LiCor, Lincoln, NE).

### Cytokine expression assay

A possible alteration in cytokine expression for Everolimus treated CAFs was measured by a human cytokine array system (Proteome Profiler™ Array, Human Cytokine Array Panel A, R&D Systems Europe, Ltd., UK & Europe). Expression of the following cytokines were determined: CD 40 Ligand, G-CSF, GM-CSF, CROa, I-309, sICAM-1, IFN-γ, IL-1a, IL-1ß, IL-1ra, IL-2, IL-6, IL-8, IL-13, IL-16, IL-17, IL-17E, IL-23, MCP-1, MIF, Serpin E1. CAFs were treated with 1 μmol Everolimus for 10 min. The Everolimus treated media and a sample of untreated CAFs-media were incubated with 15 μL of reconstituted Human Cytokine Array Panel A Detection Antibody Cocktail for one hour. Subsequently the incubated media was added to a buffer-prepared 4-Well Multi-dish and incubated for 12 h. After blotting the media/antibody solution on specific membranes, these membranes were exposed to an X-ray film to visualise the extent the different cytokines expression. Changes of cytokine expression between Everolimus treated and untreated CAFs-media were measured by densitometry (Image J 1.41o, National Institute of Health, USA).

### Statistical analysis

Data was analysed using SPSS for Macintosh (Version 21.0) software (IBM Corporation, New York, USA). All metric parameters are expressed as total numbers (%) or mean ± standard deviation (SD). Comparison between groups was made using an unpaired *t*-test. A *p*-value <0.05 was considered statistically significant.

## Results

### Characterization and immunocytochemical staining of collected CAFs

**Fig. 1:**
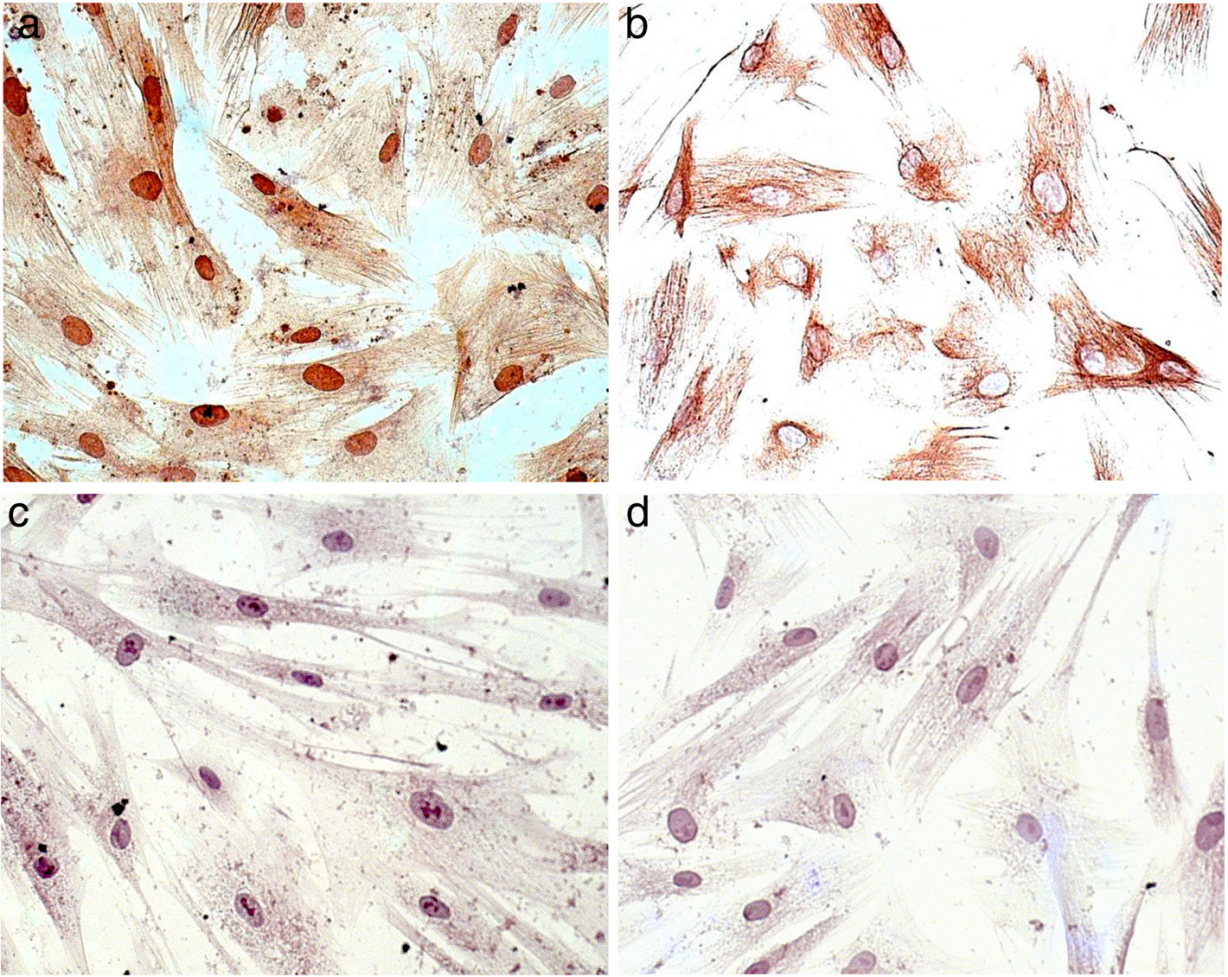
**a**-**d**. Immunocytochemical staining of collected CAFs. Immunocytochemical staining for α-smooth muscle actin (**a**), Vimentin (**b**), pan-cytokeratin marker (**c**) and HE (**d**) as negative control group

Adherent cells stained strongly for both α-smooth muscle actin (Fig. 1a) and Vimentin (Fig. 1b) and were negative for the pan-cytokeratin marker KL-1 (Fig. 1c). Hemalaun eosin staining of adherent cells displayed elongated morphology characteristic of fibroblast cells (Fig. 1d).

### Effect of immunosuppressive and cytostatic agents on tumour cell-viability

**Fig. 2:**
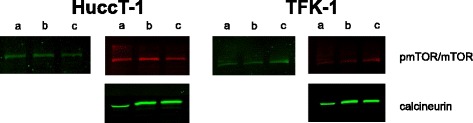
Fluorescence Western-blot for target proteins. Fluorescence Western-blot for the detection of the target proteins mTOR for Everolimus and calcineurin for CSA after 24 h (a), 48 h (b) and 96 h (c) in DMEM solution

**Fig. 3:**
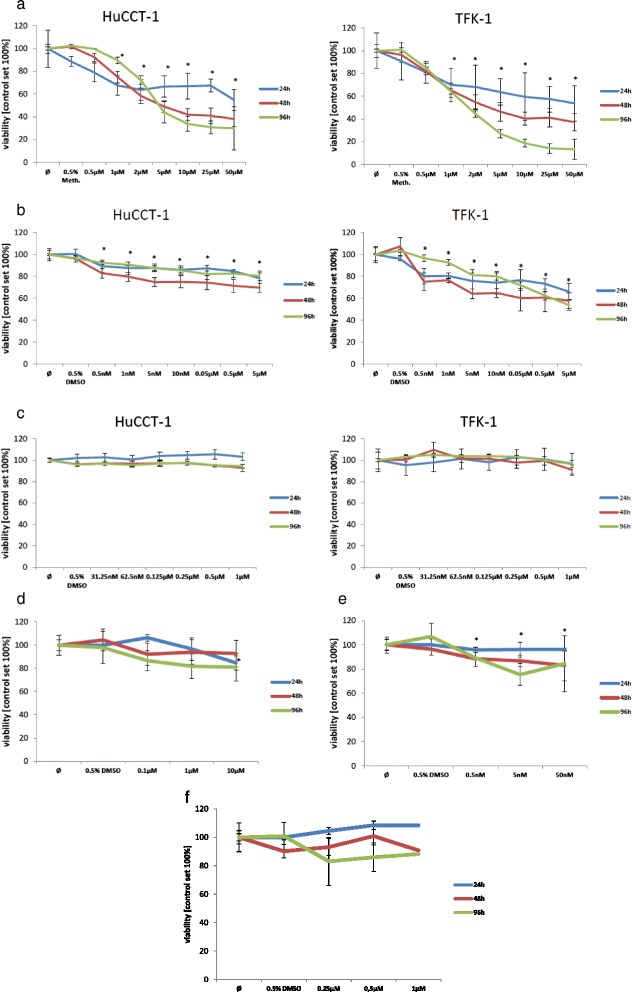
**a** Proliferation of HuCCT-1 and TFK-1 under treatment with MPA, *p <0.05, unpaired *t*-test. Tumour cells were treated with DMEM plus 10 % FCS containing different concentrations of MPA. Cell viability was measured after 24, 48 and 96 h using Crystal violet assay (blue line: 24 h of treatment with different drug concentrations of MPA, reed line: blue line: 48 h of treatment with different drug concentrations of MPA, green line: 96 h of treatment with different drug concentrations of MPA). **b** Proliferation of HuCCT-1 and TFK-1 under treatment with Everolimus, *p <0.05, unpaired *t*-test. Tumour cells were treated with DMEM plus 10 % FCS containing different concentrations of Everolimus. Cell viability was measured after 24, 48 and 96 h using Crystal violet assay (blue line: 24 h of treatment with different drug concentrations of Everolimus, reed line: blue line: 48 h of treatment with different drug concentrations of Everolimus, green line: 96 h of treatment with different drug concentrations of Everolimus). **c** Proliferation of HuCCT-1 and TFK-1 under treatment with CSA. Tumour cells were treated with DMEM plus 10 % FCS containing different concentrations of CSA (blue line: 24 h of treatment with different drug concentrations of CSA, reed line: blue line: 48 h of treatment with different drug concentrations of CSA, green line: 96 h of treatment with different drug concentrations of CSA). **d** Proliferation of CAFs under treatment with MPA, *p <0.05, unpaired *t*-test. CAFs were treated with DMEM plus 10 % FCS containing different concentrations of MPA. Cell viability was measured after 24, 48 and 96 h using Crystal violet assay (blue line: 24 h of treatment with different drug concentrations of MPA, reed line: blue line: 48 h of treatment with different drug concentrations of MPA, green line: 96 h of treatment with different drug concentrations of MPA). **e** Proliferation of CAFs under treatment with Everolimus, *p <0.05, unpaired *t*-test. CAFs were treated with DMEM plus 10 % FCS containing different concentrations of Everolimus. Cell viability was measured after 24, 48 and 96 h using Crystal violet assay (blue line: 24 h of treatment with different drug concentrations of Everolimus, reed line: blue line: 48 h of treatment with different drug concentrations of Everolimus, green line: 96 h of treatment with different drug concentrations of Everolimus). **f** Proliferation of CAFs under treatment with CSA. CAFs were treated with DMEM plus 10 % FCS containing different concentrations of CSA. Cell viability was measured after 24, 48 and 96 h using Crystal violet assay (blue line: 24 h of treatment with different drug concentrations of CSA, reed line: blue line: 48 h of treatment with different drug concentrations of CSA, green line: 96 h of treatment with different drug concentrations of CSA)

To study the effect of the different drugs, we first treated the two tumour cell lines and CAFs with variable concentrations of CSA, Everolimus and MPA. The target proteins mTOR and calcineurin were detected by fluorescent Western-Blot analysis in both tested cell lines, TFK-1 and HUCCT-1, at the different time points of cell-viability measurement (Fig. 2). Analysis of cell viability revealed that MPA induced a strong dose and time dependent effect on tumour cell lines (Fig. 3a). For HUCCT-1 and TFK-1 cells a significant lower viability was measured for every tested dosage at 24 h, 48 h and 96 h of treatment except the lowest treatment dose of 0.5 μM for 24 h (*p* < 0.05, unpaired *t*-test). Compared to MPA, Everolimus showed a weaker dose and time dependent effect although after 96 h of treatment this difference became less obvious (Fig. 3b). A significant lower viability was nevertheless measured for both tested cell lines with Everolimus treatment for every tested dosage at 24 h, 48 h and 96 h of treatment (*p* < 0.05, unpaired *t*-test). CSA showed no significant influence on cell viability, even at high concentrations (Fig. 3c). For CAFs a dose and time dependent effect was observed only for the treatment with Everolimus. A significant lower viability was measured with 0.5 nM, 5 nM and 50 nM at 48 h and 96 h of treatment (*p* < 0.05, unpaired *t*-test) (Fig. 3d). MPA and CSA showed no significant influence on cell viability of CAFS in a dose dependent manner except for the test dosage of 10 μM at 24 h with MPA treatment (*p* < 0.05, unpaired *t*-test) (Fig. 3e and f).

### Effect on tumour-cell migration after treatment with Everolimus, MPA and CSA in co-culture

**Fig. 4:**
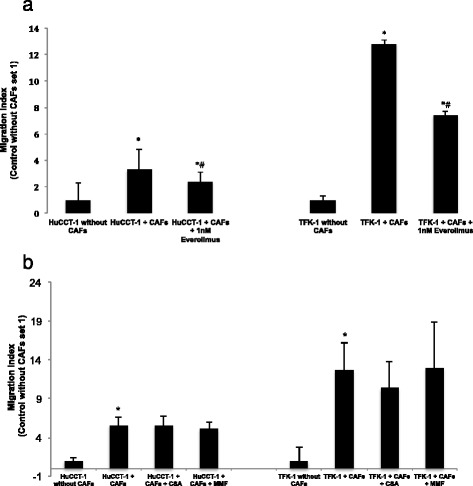
**a** Modified co-culture transmigration assay for HuCCT-1/CAF and TFK-1/CAF treated with Everolimus. *Non-treated co-culture vs. monoculture, #With 1 nM Everolimus treated co-culture vs. non treated co-culture: *p* <0.05, unpaired *t*-test. **b** Modified co-culture transmigration assay for HuCCT-1/CAF and TFK-1/CAF treated with CSA or MPA. The used drug concentrations were 0.25 μM for CSA and 0.1 μM for MPA. *Non-treated co-culture vs. monoculture: *p* <0.05, unpaired *t*-test

We next analysed CAF-mediated migratory activity of TFK-1 and HuCCT-1 in a co-culture system. Boyden chamber assays demonstrated that isolated CAFs stimulated the migratory and proliferative potential of the intrahepatic CCA cell lines. We noticed a significantly higher migratory activity for both CCA-cell lines with CAFs in co-culture (TFK-1/CAFs: 6.3 (±3.5) cells/0.35 mm^2^, *p* = 0.00014; HuCCT1/CAFs: 29.8 (±1) cells/0.35 mm^2^, (*p* = 0.001) compared to the CCA-cell line monoculture (TFK-1: 0.5 (±1.7) cells/0.35 mm^2^; HuCCT1: 5.3 (±0.4) cells/0.35 mm^2^). We then performed the test in the presence of Everolimus, MPA or CSA. A dosage of 0.25 μM was used for CSA, 0.1 μM was used for MMF and 1 nM was used for Everolimus treatment. Compared to the untreated co-culture (TFK-1/CAFs: 21.3 (±2) cells/0.35 mm^2^; HuCCT1/CAFs: 51.3 (±8.9) cells/0.35 mm^2^), the Everolimus treated co-culture showed a significant inhibition of tumour cell migration for both treated CCA-cell-lines (TFK-1/CAFs: 12.3, (±2) cells/0.35 mm^2^, *p* = 0.000013; HuCCT1/CAFs: 36.8, (±5.3) cells/0.35 mm^2^, *p* = 0.009) (Fig. 4a). For MPA and CSA no significant effect was measured (Fig. 4b).

### Western blot analysis determining cell activation and presence of drug target proteins

**Fig. 5:**
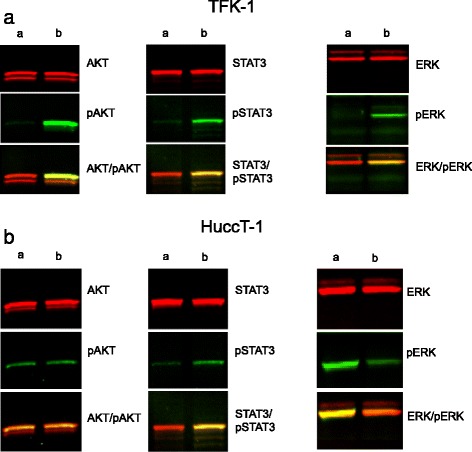
**a**. Fluorescence Western-blot for STAT3-, AKT- and ERK-pathways in CCA-cell line TFK-1. Cell-line in DMEM-media (a) and after stimulation by conditioned CAF-media for 15 min (b). **b** Fluorescence Western-blot for STAT3-, AKT- and ERK-pathways in CCA-cell line HuCCT-1. Cell-line in DMEM-media (a) and after stimulation by conditioned CAF-media for 15 min (b)

**Fig. 6:**
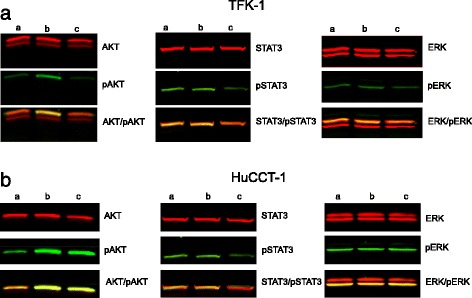
**a**. Fluorescence Western-blot after treatment of the TFK-1 tumour cell line by Everolimus. Fluorescence Western-blot was processed after treatment with Everolimus for 24 h and a following stimulation by conditioned CAF-media and 100 ng/mL hIL6 in 0 % FCS DMEM for 15 min ((a): cell-line in DMEM-media, (b): treatment with 10 nM Everolimus, (c): treatment with 1 μM Everolimus). **b**. Fluorescence Western-blot after treatment of the HuCCT-1 tumour cell line by Everolimus. Fluorescence Western-blot was processed after treatment with Everolimus for 24 h and a following stimulation by conditioned CAF-media and 100 ng/mL hIL6 in 0 % FCS DMEM for 15 min ((a): cell-line in DMEM-media, (b): treatment with 10 nM Everolimus, (c): treatment with 1 μM Everolimus)

After stimulating cancer cells with conditioned fibroblast media, the level of phosphorylated STAT3, AKT and ERK were observed to increase noticeably in the TFK-1 cell line. In the HuCCT-1 cell line only an up-regulation of phosphorylated STAT3 was observed. Highest levels were detected fifteen minutes after stimulation with conditioned media (Fig. 5a, b). Pre-treatment of TFK-1 cells with 1 μM Everolimus for 24 h followed by stimulation with conditioned CAF-media resulted in a decrease of STAT3-phosphorylation and slight decrease in AKT-phosphorylation. A decrease in phosphorylated ERK-kinase was not noticed for this cell line (Fig. 6a). With the HUCCT-1 cell line we only observed a decrease of the STAT3-phosphorylation with 1 μM Everolimus pre-treatment but surprisingly no decrease in levels of phosphorylated ERK- and AKT-kinase was observed (Fig. 6b). Treatment of the tumour cell lines with Everolimus-treated CAFs conditioned media showed no effect on phosphorylation status for either of the two cell lines.

### Analysis of cytokine expression by cholangiocarcinoma-CAFs

**Fig. 7:**
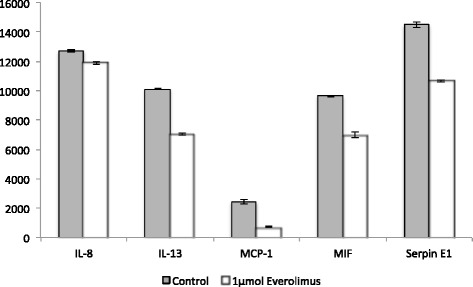
Densitometric measurement of cytokine expression by CCA-CAFs. CAFs treated with 1 μM Everolimus for 10 min show a significant lower cytokine expression compared to non-treated CAFs (*p* <0.05, unpaired *t*-test)

Having observed a significant inhibition of tumour cell migration for the Everolimus treated co-culture, the influence of Everolimus treatment on CAF-cytokine expression was measured using the human cytokine array to investigate the influence of the drug on paracrine tumour-cell stimulation. A significant lower cytokine expression for Everolimus treated CAFs was detected by densitometric evaluation. This significant lower cytokine expression was measured after 10 min of treatment with 1 μM Everolimus for cytokines IL-8 (*p* = 0.035), IL-13 (*p* = 0.0012), MCP-1 (*p* = 0.035), MIF (*p* = 0.03) and Serpin E1 (*p* = 0.02) (Fig. 7). The expressions of the other 16 tested cytokines were not significantly affected.

## Discussion

We investigated the influence of primary cultured CAFs obtained from CCA-tumour resections on tumour migration and proliferation of an intrahepatic and extrahepatic CCA cell line. We specifically analysed the influence of the mTOR-inhibitor Everolimus and MPA in a human in-vitro CAF-CCA tumour cell co-culture model. The major finding of the study was that a concurrent inhibition on tumour cell proliferation and migration occurs following Everolimus treatment. Several independent observations indicate that inhibition of migration was likely mediated by a decrease in paracrine stimulation of tumour cells by CAFs. Firstly, no changes in phosphorylated STAT3, AKT and ERK where observed in CCA cell lines at concentration which reduced migration. Secondly, Everolimus treatment of CAFs resulted in a decrease in CAF-secreted cytokines which are known to promote tumour cell migration. The rational for this project was to test whether immunosuppressive drugs that are crucial to successfully treat patients who underwent liver transplantation can also be of use in anti-cancer treatment. The results of the study open new possibilities for integrating agents that were traditionally avoided, into anti-cancer treatment protocols in the systemic treatment of recurrent malignancies after solid organ transplantation.

In this study we were able to show that the immunosuppressive drugs Everolimus and especially MPA, have an inhibitory effect on proliferation of CCA tumour cells. The strong antiproliferative effect of MPA was seen for both tumour cell lines. Previously, other groups reported similar effects in cell lines derived from other cancer types including multiple myeloma, leukemia, lymphoma, Walker’s carcinosarcoma, glioblastoma, pancreatic, lung and colon [26–30]. These groups reported a strong significant antiproliferative effect following MPA-treatment [26–31, 37] and less significant antiproliferative effects for mTOR-inhibitor-treatment [14, 15]. For Everolimus antiproliferative effect was attenuated after 96 h compared to shorter duration of treatment. This observed effect might be related to the half-life of this drug, which is known to be 30 h.

Both CCA cell-lines revealed enhanced migration under co-culture conditions with CAFs, similar to other tumour cells of different cancer types [34, 38–45]. It is well known, that CAFs promote tumour progression through the secretion of various growth factors and cytokines leading to paracrine activation of numerous intracellular signalling pathways [46]. After stimulation of the TFK-1 cell line by conditioned CAF-media we observed increased phosphorylation of STAT3, AKT and ERK in the Western blot analysis. In contrast conditioned CAF-media stimulation of HuCCT-1 cell line only resulted in activation of the JAK/STAT3-pathway. These findings are consistent with a previous study, in which activation of STAT3 by CAFs-secreted IL-22 was demonstrated in gastric cancer cells [47]. However, another study showed activation of PI3K/AKT and MAPK/ERK in the presence of CAFs [48]. Furthermore, a study group from Japan demonstrated activation of ERK1/2 and AKT pathways by conditioned media from hepatic stellate cell cultures in cultured HuCCT-1 cells [45]. The observed lack of activation of the AKT and ERK-pathways by conditioned CAF-media in the HuCCT-1 cell line in this study compared to other studies might be related to differences in the secretion profile of the tumour supporting cell. The growth stimulatory property of supporting cells may vary depending on the cellular phenotype (CAFs vs. stellate cells) and the tissue type the supporting cell is derived from (bile duct vs. hepatic). Alternatively, observed higher proliferative activity of HuCCT-1 cell line and consequent higher metabolism might result in a higher degradation of the drug.

Treatment with Everolimus revealed significant inhibition of CAF-mediated tumour cell migration. In contrast no inhibitory effect on migration was observed with MPA treatment. The difference in the migratory response between the two drugs may be due to the differing mechanisms by which they exert their effects. Everolimus targets mTORC1 actions which primarily lead to inhibition of cell cycle progression, survival, and angiogenesis. MPA mainly inhibits proliferation via inhibition of the synthesis of guanosine nucleotides [19–22] and G1/S transition in the nucleus. A possible explanation for the ineffective inhibition of tumour cell migration by MPA might be due to a lack of inhibition of secretion of tumour cell stimulating cytokines after 30 h.

One mechanism by which tumour cell migration is inhibited is likely to be due to direct inhibition of the JAK/Stat3 pathway in CCA cells, which is primarily inhibited by Everolimus, even in conditioned CAF-media. Furthermore, it has been demonstrated by several studies that mTOR is a positive regulator of the JAK/STAT3-pathway by phosphorylation of STAT3β [49–51]. Therefore, the observed lower STAT3-activation could be a result of a reduced phosphorylation of STAT3β by the inhibited mTOR. The absence of an inhibition of the ERK-phosphorylation following an mTOR-inhibition is consistent with current literature [52, 53].

A second mechanism of inhibition of tumour cell migration is alluded to by the higher sensitivity to inhibition by Everolimus in the co-culture experiments. A reduction in migration is observed at concentrations of Everolimus which are 2000 fold less than the concentration required to reduced JAK/Stat3 phosphorylation. It is possible that cross-talk between CAFs and tumour cells leads to an additive or synergistic effect in promoting migration and this cross-talk is inhibited at the lower concentrations of Everolimus treatment. This notion is supported by the fact that CAF-cytokine secretion was significantly inhibited under Everolimus treatment. A reduced cytokine-expression by Everolimus treated CAFs was recently also described for endometrial cancer [48].

The measured cytokines in our study are known to promote tumour cell proliferation, −invasion and induce an inflammatory tumour-microenvironment. IL-13, MCP-1 and MIF have been shown to induce infiltration of immune cells and promote tumour progression, −invasion and metastasis in various cancers [54–65]. MIF, known to promote tumourigenesis by inhibiting the classic tumour suppressor gene p53 [64], also stimulates the expression of proinflammatory cytokines TNFα, interferon-γ, interleukin 1β, interleukin 6, and interleukin 8 in a positive feedback circuit [64] and therefore leads to an activation of several tumour-promoting pathways. Other important cytokines, which were significantly inhibited by Everolimus and are known to promote tumour-progression and migration via the PI3K-AKT-, JAK/STAT- and MAPK-pathways are IL-8 and Serpin E1. Serpin E1 also known as Plasminogen activator inhibitor-1 (PAI-1) regulates cell migration by modulating the pericellular proteolytic microenvironment by the JAK/STAT1 signalling pathway [66]. The cytokine IL-8 promotes the growth of various tumour types including colon, multiple myeloma and non-small cell lung cancers [67, 68]. Previous studies showed that IL-8 can trigger PI3K and MAPK pathways and induce proliferation of endothelial and non-small cell lung cancer cells [69, 70]. Assuming that CAF-secreted cytokines directly influence tumour-progression and migration and that Everolimus treatment leads to an inhibition of the CAFs’ cytokine-secretion in addition to the direct tumour cell inhibition highlights the promising therapeutic strategy utilizing Everolimus in the treatment of CCA.

One of the major advantages of this study is that we were able to isolate and cultivate α-SMA-positive CAFs from resected CCA-tumours. Previous studies [17] used stromal cells derived from non-CCA tissues. On the other hand, these isolated CAFs were more fragile and showed a compromised proliferative activity after a few passages in-vitro. One possible reason for this observation might be a higher age of the donors. Consequently, use of these CAFs for research is limited and cultivation of CAFs generated from different tumours is needed. Therefore, one bias could be a different biological behaviour of the used CAFs generated from different CCA-tumours.

## Conclusions

In conclusion we showed an antiproliferative effect of MPA and Everolimus on tumour-cell-proliferation in both CCA cell-lines. Secretion of proinflammatory cytokines by CAFs associated with activation of JAK/STAT3-, ERK- and AKT-signalling is likely be one of the major factors leading to increased migration of CCA-cells in co-culture. Treatment of CCA-cells with Everolimus partly inhibited JAK/STAT3-signaling. Furthermore, we were able to significantly reduce CAF-cytokine secretion of the tumour cell stimulating cytokines IL 8, IL 13, MCP1, MIF and Serpin E1 by the treatment with Everolimus. We propose chemotherapy in combination with Everolimus after liver transplantation as a promising therapy option for CCA.

## Abbreviations

CAFs: cancer associated fibroblasts
CCA: cholangiocellular carcinoma
IMPDH: inosine monophosphate dehydrogenase
MMF: mycophenolate mofetil
MPA: mycophenolic acid
mTOR: mammalian target of rapamycin
OLTx: orthotopic liver transplantation
